# Neural Pathways of Visual Face Recognition Immediately After Birth

**DOI:** 10.3390/life15071145

**Published:** 2025-07-21

**Authors:** Carlo Lai, Chiara Ciacchella, Daniela Altavilla, Giorgio Veneziani, Giuseppe Marano, Gaia Romana Pellicano, Giacomo Della Marca, Federico Tonioni, Paola Aceto, Marco Cecchini, Eugenio Maria Mercuri, Luigi Janiri, Marianna Mazza

**Affiliations:** 1 Department of Dynamic and Clinical Psychology, and Health Studies, Sapienza University of Rome, 00185 Rome, Italy; 2 Department of Philosophy, Communication and Performing Arts, “Roma Tre” University, 00154 Rome, Italy; 3 Unit of Psychiatry, Fondazione Policlinico Universitario A. Gemelli IRCCS, 00168 Rome, Italymariannamazza@hotmail.com (M.M.); 4 Department of Neurosciences, Università Cattolica del Sacro Cuore, 00168 Rome, Italy; 5 Unit of Neurology, Fondazione Policlinico Universitario A. Gemelli IRCCS, 00168 Rome, Italy; 6 Department of Emergency, Anesthesiological and Reanimation Sciences, Fondazione Policlinico Universitario A. Gemelli IRCCS, 00168 Rome, Italy; 7 Department of Basic Biotechnological Sciences, Intensive Care and Perioperative Clinics, University of Sacred Heart, 00168 Rome, Italy; 8 Department Women Children and Public Health, Università Cattolica del Sacro Cuore, 00168 Rome, Italy

**Keywords:** face recognition, newborns, neural correlates, neural connectivity, electroencephalographic correlates, ERPs

## Abstract

The present study aimed to investigate the electrophysiological correlates of face-identity recognition in newborn infants immediately after birth. Electroencephalographic acquisition was continuously recorded in 23 newborn infants (3 < age < 24 h of life) during the following visual task: presentation of a woman’s face for 60 s (“known face”); random presentation of 50 known faces, 50 novel women’s faces, and 50 chessboards (for 2 s each). The final sample included in ERP analyses was composed of 11 newborn infants (male/female: 6/5; age: 5 h 16′ ± 3 h 51′). A greater negative amplitude of the N290 and smaller P400 and LC2 were found in response to the known face compared with the novel one in the left hemisphere. A shorter N290 latency was detected during the known face presentation compared with the novel one, and a longer latency of the same component was observed during novel face presentation compared with the chessboard. These findings suggest that newborns process a face differently from an object at birth and that they can discriminate a new face from a familiar one previously viewed for one minute.

## 1. Introduction

Individuals’ face recognition ability is particularly important as a social cue in human beings [1], who are designed to live in wide and complex relational networks. As suggested by neuroimaging studies, humans have specific structural and functional brain areas involved in face processing and recognition [2,3,4]. In the adult brain, the ability to process faces is associated with a specific event-related potential (ERP) component, i.e., N170, localized in occipitotemporal areas [5,6,7], and its source was identified in the fusiform gyrus and superior temporal sulcus [8,9], typically found bilaterally but more reliably observed in the right hemisphere [10,11].

Consistently, several studies showed a decreased connectivity intensity among the fusiform gyrus, superior temporal sulcus, anterior temporal lobe, and occipitotemporal areas when the participants switched from the face recognition to the object recognition task [12,13]. The presence of the ability to recognize a face at birth is still a research challenge.

A recent narrative review investigated how perceptual abilities develop in humans during prenatal and perinatal life [14]. As the author claims, in the early weeks of pregnancy, senses develop in a specific order: touch, balance, chemical, hearing, and finally, sight. By 26 to 29 weeks of gestational age (GA), the auditory nerve is well developed, just before brain pathways are fully formed [15]. The vision system starts developing by 12 GA with the presence of rods and future cones, but full development of the macula and photoreceptors occurs after birth. Research shows that by 34 GA, foetuses can respond to light stimuli, showing they can process visual information even without prior experience. Specifically, experiments with light patterns indicated that foetuses seem to identify simple shapes resembling faces [16].

At birth, newborns have limited vision, struggling to see contrast, colours, and details. Their field of view is small, preventing them from noticing distant or very close objects, and they lack depth perception because they lack stereopsis. However, they can recognize nearby visual signals. Research shows that two-day-old infants can recognize shapes they have manipulated before and prefer looking at visual–tactile stimuli related to the body [14].

Regarding face recognition at birth, in the first hours of life, behavioural studies showed that newborn infants appear to be able to distinguish, memorize, and recognize a face [17,18,19,20]; in fact, newborns prefer to look at a human face-like stimulus compared with any other non-human face-like stimulus [20,21], and they prefer to look at a novel face compared with a face presented during a habituation paradigm [22,23]. These skills, together with the ability to discriminate other sensory cues (e.g., tactile stimulation, odour cues, and vocal communication signals), are fundamental in early communication between newborns and caregivers [23,24,25,26,27,28,29,30,31,32,33].

Event-related potential studies conducted with infants of two to three months of life showed the role of the N290 component (a negative peak occurring 290–350 ms after stimulus onset), located in the occipitotemporal montage, in response to face stimuli [34,35,36]. The electrophysiological characteristics of this component suggest a correspondence with the N170 [36] involved in the face perception process in adults [37,38]. In accordance, previous neuroimaging studies showed that, similarly to adults, in two-month-old infants, face stimuli elicited the activation of a wide network of cortical areas, including the fusiform gyrus and the superior temporal and inferior frontal gyri [1,39]. Moreover, in one- to five-day-old newborns, a source of activation on the bilateral posterior temporal cortex in response to a dynamic face stimulus, absent in response to moving human arms, was detected, suggesting that this activation could be a specific brain response in discriminating a communicative face compared with no face cues [40].

Regarding familiarity-related effects, ERP studies reported evidence on the posterior N290/P400 responses [41]. A longer N290 latency has been observed for habituated faces compared with novel faces in 8-month-olds [42], and a greater N290 as well as a smaller P400 amplitude were recorded for familiar compared with novel faces in 9-month-olds [43]. Similarly, Moulson and colleagues [44] reported that three-month-old infants showed a lower amplitude of P400 in occipitotemporal montages and of the negative component (400–800 ms) in frontocentral areas in response to brief in-lab exposure to the familiar face compared with the novel one. Moreover, a greater amplitude of the negative component (400–800 ms) and a lower amplitude of the late component (800–1700 ms) in response to familiar stimuli compared with unfamiliar ones were also found in six-month-old infants [45].

To date, the ability to discriminate a specific known face from an unknown face in newborn infants during the first hours of life has never been explored. Therefore, the present study aimed to investigate the electrophysiological correlates of face-identity recognition in newborn infants immediately after birth. The hypothesis was that in a sample of newborn infants, at the first hours of life, the presentation of a known face would elicit a lower N290 amplitude in the occipitotemporal montage compared with the presentation of novel faces.

## 2. Materials and Methods

### 2.1. Participants

The treatment protocol was approved by the Ethical Committee of the Fondazione Policlinico Universitario Agostino Gemelli IRCCS—Università Cattolica del Sacro Cuore, Rome, with the protocol number 8582/18 ID 1902. Infants were enrolled at the Neonatal Intensive Care Unit of Policlinico Universitario “A. Gemelli” IRCCS of Rome after obtaining signatures on written informed consent from guardians.

A total of 23 newborn infants were recruited. The inclusion criteria were birth with caesarean delivery (this criterion was selected in order to plan the timing of enrolling); 3 h < age < 24 h of life; 34 weeks < gestational age < 45 weeks; Apgar index at 1 min > 7 and at 5 min > 9; weight > 2.5 kg. The exclusion criteria were any medical problem of the newborn infants; obstetrical and neurological complications of the mother; and use of any drugs acting on the central or peripheral nervous system by the mother during the pregnancy.

### 2.2. Stimuli

The stimuli consisted of coloured digital images (336 × 365) depicting 51 different female faces (1 for the known face condition and 50 for the novel faces condition) selected from the FEI face database [46] and 1 black and white non-reversible chessboard (neutral image) (336 × 365). The known face and the chessboard stimuli were the same for all the newborn babies. The 50 different female faces were randomly presented one time each.

### 2.3. Procedure

**Figure 1:**
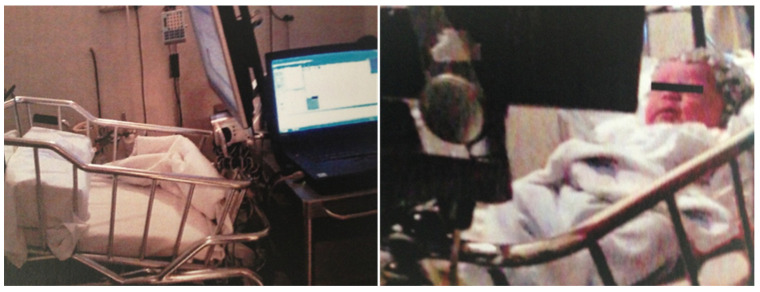
Experimental apparatus: during the visual task, the EEG (Geodesic Sensor Net 128 channels) and audio–video apparatus recorded the brain activity and the gazing behaviour of the newborn lying supine in the cradle in a quiet room.

During the hospitalization of the mother the evening before the planned caesarean birth, the researchers identified the future parents at the Neonatology Unit and asked them to sign the informed consent in order to enrol the newborn infant in the study. During the first three hours after the birth, the clinical examinations were performed by the neonatologist, and the medical staff were instructed, when possible, not to approach the newborn’s face within 20–30 cm before the experiment in order to minimize the experience with faces before the experimental test. Subsequently, the newborn infants, remaining in their cradle, were carried from the nursery to the neurophysiology laboratory by the clinicians. The experimental EEG task was performed in a soundproof room with a low and constant illumination (Figure 1). A 128-channel HydroCel Geodesic Sensor Net was placed on the head of the newborn, and then she/he was lying supine in a cradle (tilted forward at 15°) at a viewing distance of 25 cm from a monitor screen (27 cm, 75 Hz, 1024 × 768 resolution, and tilted forward 15°). A video camera was placed at the base of the cradle, below the monitor, to record the gaze of the newborns (Figure 1). Behind the newborn’s head, a mirror was placed, focused on the video camera placed below the monitor, in order to subsequently identify and exclude the trials in which the newborn infant was not looking at the monitor (Figure 1).

**Figure 2:**
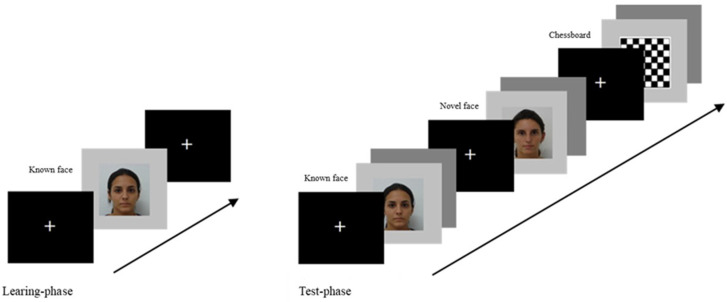
Experimental task procedure: The trial of the learning phase started with a fixation cross for 3000 ms, followed by the known face lasting 60,000 ms, and it ended with a fixation cross of 3000 ms. Subsequently, the test phase with three conditions (known face, novel faces, and chessboard) was presented. Each trial of the test phase started with the presentation of a fixation cross for 800 ms, followed by the stimulus (known face vs. novel faces vs. chessboard) lasting 2000 ms, and it ended with an inter-stimulus interval of 400/600 ms. A total of 150 trials (50 trials per condition) were presented in a random order.

The stimuli were presented at the centre of the screen on a light grey background using E-Prime (v.2.0.8.90; Psychology Software Tools, Inc., Pittsburgh, PA, USA) (Figure 2). The visual task was composed of two blocks, the learning phase and the test phase. During the learning phase, the only trial presented started with a fixation cross presented for 3000 ms, followed by the woman’s face chosen for the known face condition, lasting 60,000 ms. After the learning phase, a pause with a subsequent 3000 ms fixation cross was presented. Afterwards, the test phase started, including a randomized presentation of the three conditions (known face, novel faces, and chessboard). Each trial of the test phase started with the presentation of a fixation cross for 800 ms, followed by the stimulus (known face vs. novel faces vs. chessboard) lasting 2000 ms, and then, the trial ended with an inter-stimulus interval (grey screen) of 400/600 ms. A total of 150 trials (50 known faces vs. 50 novel faces vs. 50 chessboards) were presented in a random order (Figure 2).

### 2.4. Video Recordings Encoding

The video recordings of each newborn infant were codified, frame by frame. Video recordings of the learning phase were analysed in order to measure the familiarization process: this encoding procedure allowed us to select only the recordings in which the newborn showed a looking behaviour (opened eyes and gaze directed toward the screen) for at least 20 s.

Video recordings of the test phase were codified in order to identify and exclude the trials in which the newborn was not looking at the monitor during the stimuli presentation. This encoding procedure allowed for a selection of only the trials in which the newborn infant had opened eyes and her/his gaze was directed toward the stimulus shown on the monitor, and to exclude the trials in which the newborn infant had closed eyes or was looking away from the monitor.

### 2.5. Electroencephalographic (EEG) Recording and Event-Related Potential (ERP) Analysis

EEG data were continuously recorded at 250 Hz using NetStation 4.5.1 with 128-channel HydroCel Geodesic Sensor Net referenced to the vertex (Cz). Impedances were kept below 50 KΩ (Electrical Geodesic, Inc.; Eugene, OR). After the acquisition, in off-line mode, the data were digitally filtered at 30 Hz low-pass. The EEG data of each participant were segmented in epochs of 1100 ms duration: the segmentation was set from −100 ms before to 1000 ms after stimulus onset. The NetStation artefacts detection was set at 200 μV for bad channels (noisy electrodes), at 150 μV for eye blinks, and at 100 μV for electrodes revealing the eye movements (Electrical Geodesic, Inc., Eugene, OR, USA) [47]. Segments with an eye blink, an eye movement, or more than 20 bad channels were excluded, and bad channels replacement was performed. To ensure a minimum number of artefact-free trials for analysis, the participants with a cutoff of at least 5 artefact-free segments in all conditions were included. The data were re-referenced to average reference. The baseline was corrected at −100 ms before the stimulus onset [47,48,49]. The amplitude and the latency of ERP components were extracted automatically through Net Station software (version 4.4.2; Electrical Geodesic, Inc., Eugene, OR, USA).

Time windows for the ERP components were selected based on the literature [34,36,41,43]: between 300 and 400 ms for the N290 and between 400 and 500 ms for the P400. For the late components (LCs), three intervals were selected based on the literature [45,50,51] as follows: between 500 and 600 for the LC1; between 600 and 700 for the LC2; and between 700 and 1000 for the LC3.

After the EEG signal cleaning of artefacts, the following electrode locations were chosen for each montage: occipitotemporal montage (left electrodes 58-64-65-69-70; right electrodes 83-89-90-91-96) and frontal montage (left electrodes 23-24-27-28-34-35; right electrodes 3-110-116-117-123-124).

The ERP analyses on the occipitotemporal montage were conducted on the mean amplitude of the P200, N290, P400, LC1, LC2, and LC3 components and on the latency of N290 components. The newborn infants presenting at least five trials free from artefacts for each condition (known face, novel face, and chessboard) were included in statistical analyses.

For statistical analyses of the ERPs, data 3 × 2 repeated-measures analyses of variance (ANOVAs) with condition (known face vs. novel face vs. chessboard) and hemisphere (left vs. right) as the within-subjects factors were conducted on the amplitude and latency of each ERP component of the occipitotemporal montage. On significant effects, Fisher LSD post hoc test was performed and post hoc power analysis (1 − β) was calculated given α, partial eta-squared, and sample size.

## 3. Results

After the video encoding of the learning phase, 22 participants showed a looking behaviour (opened eyes and gaze directed toward the screen) of at least 20 s (min = 26.6 s, max = 48.0 s; mean = 40.3 s; standard deviation = 6.9 s). One newborn was excluded because she/he was asleep during the experimental task. Only the trials of the test phase in which the newborn infants were gazing at the monitor were included in the EEG data processing (known face: 30.5 ± 14.5; novel face: 30 ± 13.8; chessboard: 30 ± 14.8). After EEG data cleaning, eleven newborn infants were excluded because they did not have at least five artefact-free segments in all conditions. The final sample included in the ERP was composed of 11 newborn infants (male/female: 6/5; age of newborns: 5 h 16′ ± 3 h 51′; gestational age: 40 ± 1 weeks; first minute Apgar index: 8.8 ± 0.6; fifth minute Apgar index: 9.8 ± 0.4; weight: 3176.8 ± 336.2 g). Mean and standard deviation of the number of artefact-free trials in which the newborn infants were gazing at the monitor included in the ERPs analysis were: 15.2 ± 8.8 for the known face condition, 15.3 ± 7 for the novel face condition, and 14.7 ± 6.7 for the chessboard condition.

### ERPs

**Figure 3:**
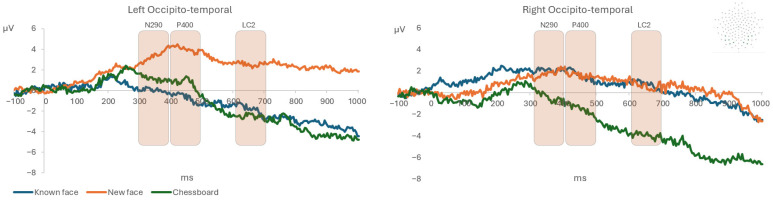
The event-related potential grand average of the left and right occipitotemporal montage in response to the known face, novel face, and chessboard.

As shown in Table 1, the 3 × 2 repeated-measures ANOVA condition (known face vs. novel face vs. chessboard) per hemisphere (left vs. right) performed on ERP amplitude and latency on occipitotemporal montage showed a significant interaction effect condition per hemisphere on the amplitude of the N290, P400, and LC2 components, in which a greater negative amplitude of N290 and a smaller amplitude of the P400 and LC2 were detected during the known face presentation compared with the novel face in the left hemisphere (Figure 3).

**Figure 4:**
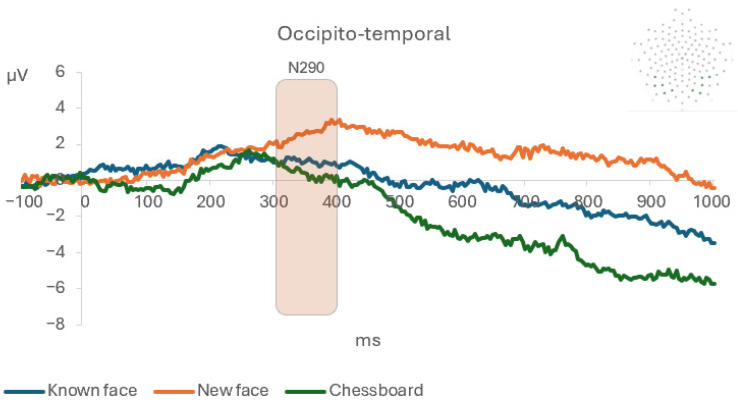
The occipitotemporal grand average for the three conditions: known face, novel face, and chessboard.

On the latency, the ANOVAs showed a significant main effect of condition on the N290 component, in which the novel face elicited a longer latency compared with the known face and the chessboard (Figure 4). No significant results were found on the frontal montage. Post hoc power analysis (1 − β) given α, partial eta-squared, and sample size showed a high statistical power (Table 1).

## 4. Discussion

The main finding of the present study was that newborn infants, in the first hours of life, showed a different neural correlate in response to a previously known face (during a static presentation of one minute) compared with a novel face and a neutral stimulus (a chessboard).

The hypothesis of the present study was that newborn infants, in the first hours of life, would present a lower N290 amplitude on the occipitotemporal montage in response to a known face compared with novel faces. The findings seem to support this hypothesis, showing that the known face elicited a shorter latency of the N290 component with a lower amplitude compared with the novel faces on the left occipitotemporal montage, and this effect was maintained continuously from 300 to 700 ms. These results appear to be consistent with previous studies [37,42,43,52], which observed a greater N290 as well as a smaller P400 amplitude for familiar compared with novel faces in 9-month-old infants, supporting the hypothesis that at the functional level, these late ERP components at birth could be associated with later-developing components such as the N170 observed in adults [53]. These results suggest that neural mechanisms for face processing already show sensitivity to familiarity in early infancy, but in components (N290 and P400) that occur later in time compared with the early N100 and P100 observed later in development [53]. The ERP amplitudes (3–6 μV) were in line with values reported in previous infant studies that showed an increase in the ERP amplitude after the age of four months [41,54,55]. This could be associated with the immaturity of the cortical structures and the incomplete myelination of nerve cells in newborn infants [41,54,55]. Another important result is that the known and novel faces showed a greater ERP amplitude of the LC2 component in the occipitotemporal area of the right hemisphere compared with the chessboard. Together with the previous result, this finding suggests that, while discriminating faces from objects seems to involve the right hemisphere, the recognition of a known face seems to involve majorly the left hemisphere. In fact, consistently with previous studies on adults, while the right hemisphere appears to be specialized in the discrimination of a face vs. an object, the left hemisphere could be more involved in memory processes, and therefore, in familiarity recognition [56,57,58,59,60,61]. Future EEG connectivity studies could test the hemispheric lateralization of the discrimination and recognition [62].

Thus, the findings of the present study suggest that newborn infants, in the first hours of life, may be able to differentiate a known face from a novel one and to distinguish a face stimulus from an object.

To date, the neural correlates of face recognition ability immediately after birth remain poorly investigated, despite seeming crucial in order to establish social competence in the first days of life. The early ability to keep in mind significant faces could be the sine qua non condition for the creation of representations and, subsequently, expectations toward the specific faces in future interactions.

Furthermore, coherently with previous ERP studies [34,37,40] that showed an effect on the N290, P400, and late components during face recognition in three-month-old and six-month-old infants, the findings of the present study seem to highlight another important point, namely, the different processing of the face compared with other objects already at birth.

This raises a crucial and discussed question about how newborn human infants could have the ability to visually discriminate a face despite the lack of experience [37,63]. Several studies have already shown [64,65] that, in human and animal babies, spontaneous preferences for faces emerge before the development of face-selective cortical domains. A possible and intriguing hypothesis could be that the foetus in the womb, through proprioception and touch (e.g., touching its own face), develops a proprioceptive face representation [16,66]. A large body of evidence has shown how, in addition to visual information, there are multiple proprioceptive, tactile, as well as motor cues that are likely to update cognitive representations of one’s face [67,68]. It could be that, after birth, the infant can visually recognize the key elements of a face (e.g., eyes, nose, mouth) using the intermodal information acquired during pregnancy through motor and proprioceptive perception of its own face [22,32,69]. If this interpretation is correct, it could raise important implications for the development of communication during intrauterine life [16,70].

The present study has some limitations. The main limitation was the small number of newborn infants included in the final sample, due to both the difficulty in recruiting newborn infants and to the presence of artefacts in the EEG data, and, overall, to the conservative criteria for the video encoding. The lack of control of the newborns’ movements, sleep, and physiological activity makes it difficult to obtain a perfectly clean EEG recording. In the present study, we tried to control the variables as much as possible by using video recordings that ensured that the newborn was looking at the stimulus, setting a minimum cutoff of artefact-free trials in each condition, and performing post hoc power analyses. However, further research with larger samples is certainly indispensable. Moreover, in future research, another important aim could be to investigate how environmental factors, such as the quality of pre-/postnatal stimulation or the mental and physical health of the mother, can modulate face recognition at birth.

In conclusion, the findings of the present study could be useful to plan future longitudinal studies in order to investigate the associations among communicative experiences with the caregiver, neural correlates, and psychological outcomes.

## Figures and Tables

**Table 1:** Significant effects of the 3 × 2 repeated-measures ANOVAs with condition (known face vs. novel face vs. chessboard) and hemisphere [left (l) vs. right (r)] as the within-subjects factors performed on amplitude and latency of event-related potential (ERP) components on occipitotemporal montage.

ERP Component	Effects on Amplitude and Latency	Fisher LSD Post Hoc [M ± SD μV and ms]
N290 (300–400 ms)	Condition per Hemisphere F(2, 20) = 3.96; *p* = 0.036, ηp^2^ = 0.28 (1 − β = 0.99)	Known face (l) [−0.11 ± 8.81] < Novel face (l) [2.67 ± 2.84] * Novel face (l) [2.67 ± 2.84] > Chessboard (r) [0.06 ± 3.34] *
	Condition F(2, 20) = 5.54; *p* = 0.012, ηp^2^ = 0.35 (1 − β = 0.99)	Known face [340.29 ± 32.91] < Novel face [363.85 ± 30.33] ** Chessboard [337.09 ± 31.00] < Novel face [363.85 ± 30.33] **
P400 (400–500 ms)	Condition per Hemisphere F(2, 20) = 5.24; *p* = 0.015; ηp^2^ = 0.34 (1 − β = 0.99)	Known face (l) [−1.18 ± 10.03] < Known face (r) [1.75 ± 7.27] * Known face (l) [−1.18 ± 10.03] < Novel face (l) [3.57 ± 3.22] ** Novel face (l) [3.57 ± 3.22] > Novel face (r) [0.44 ± 4.41] * Novel face (l) [3.57 ± 3.22] > Chessboard (r) [−0.84 ± 3.54] **
LC2 (600–700 ms)	Condition per Hemisphere F(2, 20) = 3.74; *p* = 0.042; ηp^2^ = 0.27 (1 − β = 0.99)	Known face (l) [−2.70 ± 10.77] < Novel face (l) [2.07 ± 5.19] ** Known face (r) [0.69 ± 8.31] > Chessboard (r) [−3.61 ± 3.02] * Novel face (l) [2.07 ± 5.19] > Chessboard (l) [−2.07 ± 8.43] * Novel face (l) [2.07 ± 5.19] > Chessboard (r) [−3.61 ± 3.02] ** Novel face (r) [−0.53 ± 5.53] > Chessboard (r) [−3.61 ± 3.02] *

Note: * *p*-value < 0.05; ** *p*-value < 0.01.

## Data Availability

The dataset used and analysed is available from the corresponding author on reasonable request.
